# Prediction model and demonstration of regional agricultural carbon emissions based on Isomap–ACO–ET: a case study of Guangdong Province, China

**DOI:** 10.1038/s41598-023-39996-5

**Published:** 2023-08-04

**Authors:** Yanwei Qi, Huailiang Liu, Jianbo Zhao

**Affiliations:** School of Economics and Management, Xidian University, Xi’an, 710071 China

**Keywords:** Environmental impact, Environmental impact

## Abstract

Scientific analysis of regional agricultural carbon emission prediction models and empirical studies are of great practical significance to the realization of low-carbon agriculture, which can help revitalize and build up ecological and beautiful countryside in China. This paper takes agriculture in Guangdong Province, China, as the research object, and uses the extended STIPAT model to construct an indicator system for the factors influencing agricultural carbon emissions in Guangdong. Based on this system, a combined Isomap–ACO–ET prediction model combing the isometric mapping algorithm (Isomap), ant colony algorithm (ACO) and extreme random tree algorithm (ET) was used to predict agriculture carbon emissions in Guangdong Province under five scenarios. Effective predictions can be made for agricultural carbon emissions in Guangdong Province, which are expected to fluctuate between 11,142,200 tons and 11,386,000 tons in 2030. And compared with other machine learning and neural network models, the Isomap–ACO–ET model has a better prediction performance with an MSE of 0.00018 and an accuracy of 98.7%. To develop low-carbon agriculture in Guangdong Province, we should improve farming methods, reduce the intensity of agrochemical application, strengthen the development and promotion of agricultural energy-saving and emission reduction technologies and low-carbon energy sources, reduce the intensity of carbon emissions from agricultural energy consumption, optimize the agricultural planting structure, and develop green agricultural products and agro-ecological tourism according to local conditions. This will promote the development of agriculture in Guangdong Province in a green and sustainable direction.

## Introduction

The data show that human activities have caused an increase in global greenhouse gas emissions since the industrial era, with a 70 per cent increase between 1970 and 2021^1,2^. Carbon dioxide, on the other hand, is the most important anthropogenic greenhouse gas, with emissions increasing by about 80 per cent between 1970 and 2021^3,4^. The bulletin released by the World Meteorological Organization shows that the global average annual temperature has increased by 0.7 °C during the 100-year period from the end of the nineteenth century to the end of the twentieth century, and climate change caused by greenhouse gases has become a serious challenge shared by all mankind in the twenty-first century^5,6^. The study of the United Nations Intergovernmental Panel on Climate Change (IPCC) shows that greenhouse gases generated by agricultural activities account for 13.5% of the total global greenhouse gas emissions^7,8^. And according to the Food and Agriculture Organization of the United Nations (FAO), "agriculture, forestry and land-use change account for about 20% of global greenhouse gas emissions"^9,10^. From these data, it is clear that agriculture is an important source of GHG emissions. The increasing carbon emissions have an extremely significant impact on global climate change, and the increase in carbon emissions due to human behaviour is one of the major causes of global warming^11,12^. China has also played an important role in combating the global climate change and pledged in the Copenhagen Conference to reduce its carbon dioxide emissions per unit of GDP by 60–65 per cent in 2030 compared to 2005^13,14^.

A growing number of scholars at home and abroad have conducted many studies on agricultural carbon emissions, mainly focusing on six aspects. Firstly, measurement of agricultural carbon emissions. For example, Wang et al.^15^ measured the total amount and intensity of China’s agricultural carbon emissions from 2000 to 2016 and compared the regional differences in China’s agricultural carbon emissions. Chen et al.^16^ remeasured agricultural carbon emissions in Fujian Province, China, from 2008–2017 based on five major sources of carbon emissions from agriculture. Secondly, exploration of the drivers of agricultural carbon emissions. For example, Xiong et al.^17^ used the STIRPAT model to identify the factors influencing agricultural carbon emissions in Jiangsu Province, China, and concluded that the influencing factors were: agricultural production efficiency, agricultural structure, agricultural economic development level, agricultural population size, urbanization, mechanization and the degree of natural disasters. Yang et al.^18^ studied the drivers of agroecological efficiency growth at the provincial level in China and concluded that the drivers were: technological progress, agricultural infrastructure, improvements in human capital and public investment. Thirdly, analysis of the spatial and temporal characteristics of agricultural carbon emissions. For example, Hu et al.^19^ measured and analyzed the spatial and temporal characteristics of agricultural carbon emissions in Jiangsu Province, China, from three carbon sources: agricultural land use, rice cultivation and livestock farming. Zhou et al.^20^ calculated the agricultural carbon emissions in China’s "Belt and Road" region from 2003 to 2018 and analyzed their spatial and temporal characteristics. Fourthly, study of the interaction between agricultural carbon emissions and economic development. For example, Zang et al.^21^ conducted a study on the relationship between agricultural carbon emission intensity, agricultural economic development and agricultural trade in China from 2002 to 2020. Shu-jie et al.^22^ analyzed the decoupling relationship between agricultural carbon emissions and economic growth in Jilin Province, China, from 1999 to 2014. Fifthly, efficiency measures of agricultural carbon emissions. For example, Wang and Feng^23^ evaluated the efficiency of China’s agricultural carbon emissions from both static and dynamic perspectives based on DEA and Theil models. Zhang et al.^24^ used the SBM-DEA model to measure the economic efficiency of inter-provincial agriculture in China for the years 2000–2019. Sixthly, study of carbon reduction measures in agriculture. For example, Panchasara et al.^25^ explored the main sources of carbon emissions from the agricultural sector in Australia and review effective ways to reduce greenhouse gas emissions through smart farming technologies. Cui et al.^26^ studied agricultural panel data from 31 provinces in mid-1997–2017 and suggested differentiated carbon reduction policies based on regional and temporal differences, as well as a carbon reduction market trading mechanism and compensation policies. In summary, it can be seen that scholars at home and abroad have made fruitful achievements in the research of agricultural carbon emissions, but there are fewer studies related to the prediction and empirical evidence of regional agricultural carbon emissions, and the exploration is still insufficient.

The research of the above scholars has contributed to the measurement of agricultural carbon emissions, drivers of agricultural carbon emissions, analysis of the spatial and temporal characteristics of agricultural carbon emissions, the interaction between agricultural carbon emissions and economic development, the efficiency measurement of agricultural carbon emissions, and agricultural carbon emission reduction measures, which have made the research on agricultural carbon emissions more and more rich and in-depth. However, there is no research on the prediction of agricultural carbon emissions, especially on the prediction of regional agricultural carbon emissions using methods such as machine learning. In this paper, the prediction of regional agricultural carbon emissions will be investigated using a machine-learning based combined prediction model and empirically demonstrated taking Guangdong Province’s agriculture as the research object.

The contribution of this paper lies in the selection of seven variables such as the number of agricultural populations, GDP per capita of rural inhabitants and energy efficiency of agricultural production as factors influencing regional agricultural carbon emission. This paper also establishes a combined Isomap–ACO–ET prediction model combing the Isomap algorithm (Isomap), Ant Colony Algorithm (ACO), and Extreme Random Tree Algorithm (ET) based on the indicator data for the period of 2000–2020 to predict the regional agricultural carbon emissions in Guangdong Province under five scenarios. The results show that regional agricultural carbon emissions in Guangdong Province generally showed an upward and then a downward trend during the period of 2000–2021.

The innovations of this paper are as follows:

1. For the prediction of agricultural carbon emissions, a combined prediction model based on machine learning is given, and the validity of the model is verified by empirical evidence on the agricultural data of Guangdong Province.
2. The combined prediction model proposed in this paper is applied to predict the agricultural carbon emissions of Guangdong Province’s agriculture in the next nine years under multiple scenarios. It is expected that the agricultural carbon emissions of Guangdong Province in 2030 will fluctuate between 11,142,200 tons and 11,386,600 tons.
3. The machine learning combinatorial prediction model proposed in this paper has a better prediction accuracy on the agricultural data of Guangdong Province compared with other models, with a mean square error of 0.00018 and an accuracy rate of 98.7%.

Guangdong Province is located in the southern part of mainland China, with an average annual sunshine of more than 1800 h, a total annual radiation of more than 5.9 × 105 J/cm2, an average annual precipitation of 1652.5 mm. Guangdong Province has a high topography in the north and a low topography in the south, and is rich in water resources. Such geographical and climatic conditions provide favorable conditions for agriculture, making Guangdong Province one of the most productive regions in China^27^. The current type of agricultural development in Guangdong Province belongs to the typical chemical agriculture that overly relies on high-carbon production means such as chemical fertilizers and pesticides^28^. Therefore, this paper studies regional agricultural carbon emissions in Guangdong Province to provide a reference for the implementation of green agricultural development and agricultural carbon emission reduction policies in Guangdong Province agriculture, which will lead to the realization of low-carbon agriculture and overall emission reduction targets.

## Methods

The Isometric Mapping Algorithm (ISOMAP) is based on geometric distances instead of traditional Euclidean geometric distances, and then uses Multidimensional Scaling (MDS) algorithms to isometrically embed the dataset isometrically from the high-dimensional space into the lowdimensional space, thus obtaining a low-dimensional matrix of samples with constant geodetic distances between the sample data^29^.

The Euclidean distance (Euclidean metric) is the most common representation of the distance between two or more points, defined in Euclidean space, for any n-dimensional vector $x=({x}_{1}, {x}_{2}, \dots , {x}_{n})$ and $y=({y}_{1}, {y}_{2}, \dots , {y}_{n})$, whose distance $d\left(x,y\right)$ is calculated as follows:

1 $$d\left(x,y\right)= \sqrt{\sum_{i=1}^{n}{({x}_{i}-{y}_{i})}^{2}}$$

The geodesic distance model focuses on the spatial distribution of the data, and characterizes the spatial distance between two points by searching for the shortest path, which can reflect the spatial distance between two points more realistically. The geodesic distance calculation method uses the nearest neighbor algorithm to construct the nearest neighbor graph and searches for the shortest path in the nearest neighbor graph based on the shortest path algorithm.

The Extreme Random Trees (ET) algorithm is based on the Random Forest (RF) algorithm and can be used for non-linear system modelling and regression prediction^30^. Unlike the RF algorithm, which uses Bootstrap to extract the training set, the ET algorithm uses all training sets when training the decision tree, which has relatively stronger generalization capability. For the collected data set $\Omega = \{({A}_{1}, {y}_{1}), ({A}_{2},{ y}_{2}) ... ({A}_{N},{ y}_{N})\}$, where ${A}_{i}$ is a 1 × 5-dimensional row vector, a set of inputs for the modelled samples, ${y}_{i}$ is the true output value of the sample corresponding to ${A}_{i}$, $i= 1, 2 ... N$, $N$ is the number of sample groups, the ET algorithm is used to train the data based on the Training.

Ant Colony Optimization (ACO) is a heuristic search algorithm based on population search^31^. In the ACO algorithm let, there are a total of m food source nodes, and the set of all food sources that each ant can find is $s (s=1, 2, ..., m)$. The distance between any two nodes on the food source is $d$, and the amount of residual information between two nodes at any moment $t$ is $\tau (t)$. At the start of the algorithm, the amount of information between any two food source nodes is the same, i.e. $\tau (0) = c$ ($c$ is a very small constant). The taboo table ${Tabu}_{\xi } (\xi = 1, 2..., n)$ in the ACO algorithm is used to denote the set of all paths taken by the ${\xi }^{th}$ ant in its search for the food source node, and the first element of the taboo table is set to the current position of the ${\xi }^{th}$ ant. Thus, the probability of an ant walking from the ${i}^{th}$ food source node to the ${j}^{th}$ food source node at moment $t$, ${p}_{ij}^{\xi }\left(t\right)$ is as follows:

2 $$ p_{ij}^{\xi } \left( t \right) = \left\{ {\begin{array}{*{20}l} {\frac{{\left[ {\tau_{ij} \left( t \right)} \right]^{\alpha } \left( {\eta_{ij} } \right)^{\beta } }}{{\mathop \sum \nolimits_{{s \in J_{\xi }^{\left( i \right)} }} \left[ {\tau_{i\xi } \left( t \right)} \right]^{\alpha } \left( {\eta_{j\xi } } \right)^{\beta } }},} \hfill & {j \in J_{k\xi }^{\left( i \right)} } \hfill \\ {0,} \hfill & {others} \hfill \\ \end{array} } \right. $$

where ${J}_{k\xi }^{(i)} = \{S-{tabu}_{\xi }\}$ denotes the food source node to be selected next by the ${\xi }^{th}$ ant; $\eta$ denotes the heuristic information of the path; α denotes the relative importance of the amount of information on the path; $\beta$ denotes the relative importance of the heuristic information.

The Isomap–ACO–ET model is a combination of the above three algorithms. That is, Isomap–ACO–ET model is a combination of the Isometric Mapping Algorithm (ISOMAP), Ant Colony Algorithm (ACO) and Extreme Random Tree Algorithm (ET). Here, we first use the Isomap algorithm to dimensionality reduce the agricultural start data of Guangdong province from 2000 to 2021 to get the new data after dimensionality reduction, and then split the new data into a training set and a test set, and input the training set into the ET algorithm for training. Then, the ACO algorithm is used to help find the optimal hyperparameters of the ET algorithm during the training process. The test set is input into the optimal ET model for testing to test the predictive validity of the model. Finally, the ET algorithm with optimised hyperparameters is combined with the previous Isomap and ACO, Isomap–ACO–ET model.

In this paper, the Isomap–ACO–ET combined forecasting model is applied to forecast the carbon emissions of Guangdong Province from 2022 to 2030 under multiple scenarios, and the combined forecasting model is compared with other models to verify the performance advantages of the combined forecasting model over other models.

The Scalable Stochastic Environmental Impact Assessment (STIRPAT) model is derived from the IPAT equation, based on which, York et al.^32^ constructed the STIRPAT model with the expression:

3 $$I=a{P}^{b}{A}^{c}{T}^{d}\mu $$

where $I$ is the environmental load; $P$ is the population size; $A$ is the affluence; and $T$ is the technology level. $a$ is the constant term; $b$, $c$ and $d$ are the exponential terms for $P$, $A$ and $T$, respectively; and $\mu$ is the error term.

The STIRPAT model can quantitatively analyze the effects of factors on environmental loads, and the method has been widely used in environmental protection studies. In the process of using the STIRPAT model to study carbon emissions, it is possible to construct an extended STIRPAT model based on the actual situation of the study area by introducing other factors that can have an impact on carbon emissions. In order to reflect the influence of economic development and technological progress on agricultural carbon emissions, this paper selects seven factors as independent variables to extend the STIRPAT model, including the number of agricultural population $(P)$, agricultural GDP per capita $(A)$, the level of agricultural mechanization $(T)$, agricultural production energy efficiency $(E)$, agricultural industry structure $(S)$, disposable income of rural residents $(N)$, and the area of mechanized arable land $(J)$. The extended model was constructed as follows:

4 $$I=a{P}^{b}{A}^{c}{T}^{d}{E}^{e}{S}^{f}{N}^{g}{J}^{h}\mu $$

where $b$, $c$, $d$, $e$, $f$, $g$ and $h$ are the index terms for $P$, $A$, $T$, $E$, $S$, $N$ and $J$ respectively.

In order to eliminate possible heteroskedasticity effects in the model, the study logarithmic all variables and the extended STIRPAT model after legitimization is as follows:

5 $$lnI=lna+ blnP+ clnA+ dlnT+elnE+flnS+glnN+hlnJ+ln\mu $$

The number of agricultural populations $(P)$, agricultural GDP per capita $(A)$ and seven other indicators affecting agricultural carbon emissions in Guangdong Province were selected for this paper.

Since the units of the individual indicators in the embodiment of the selected indicators vary, in order to eliminate the influence of the data scale on the prediction effect of the model, it is necessary to standardize the original data to be dimensionless. In this paper, the mean-standard deviation standardization method is used, and the standardized data of the independent and dependent variables are denoted as ${z}_{1}, {z}_{2},...,{ z}_{7}$ and $\widetilde{y}$ respectively. The process of data standardization is:

6 $${Z}_{i}= \frac{{X}_{i}- \mu }{S}$$

where ${Z}_{i}$ is the normalized data, ${X}_{i}$ is the original data, $\mu$ is the mean, and $S$ is the standard deviation.

### Empirical analysis

The data on the number of agricultural population $(P)$, agricultural GDP per capita $(A)$, level of agricultural mechanization $(T)$, agricultural industrial structure $(S)$, energy efficiency of agricultural production $(E)$, area of mechanically cultivated arable land $(J)$, disposable income of rural residents $(N)$ and total agricultural carbon emissions $(I)$ required for agricultural carbon emissions in Guangdong Province from 2000 to 2021 were obtained from the previous years of Guangdong Statistical Yearbook, China Rural Statistical Yearbook and China Agricultural Yearbook. In order to exclude inflation and other price increases, per capita agricultural GDP and disposable income of rural residents were converted at constant prices in 2000.

**Figure 1:**
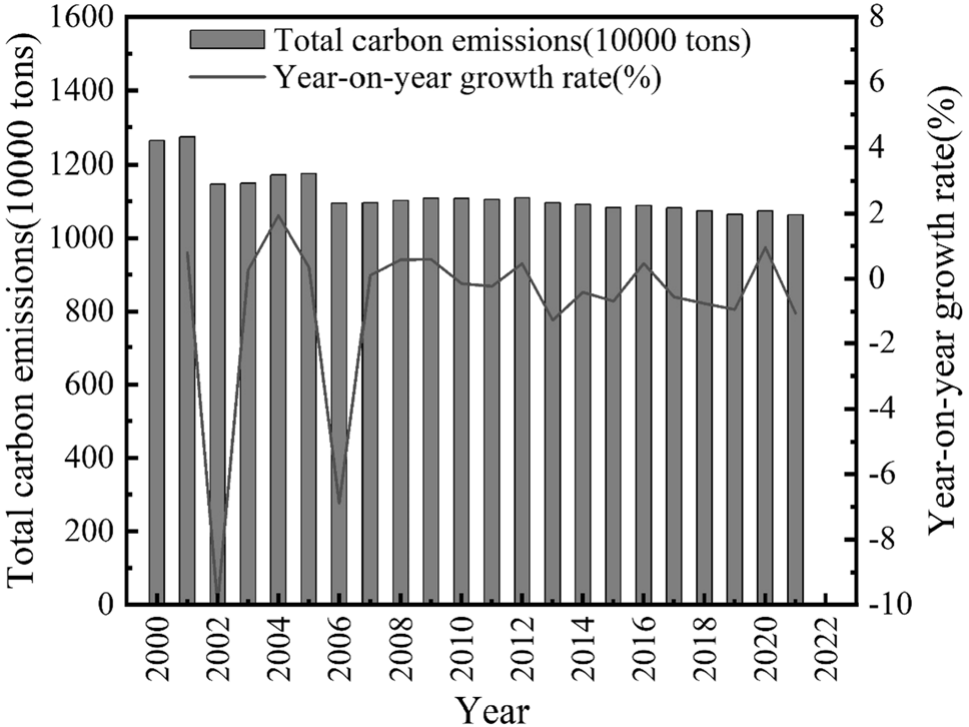
2020–2021 Agricultural carbon emission and annual growth rate of Guangdong Province.

**Figure 2:**
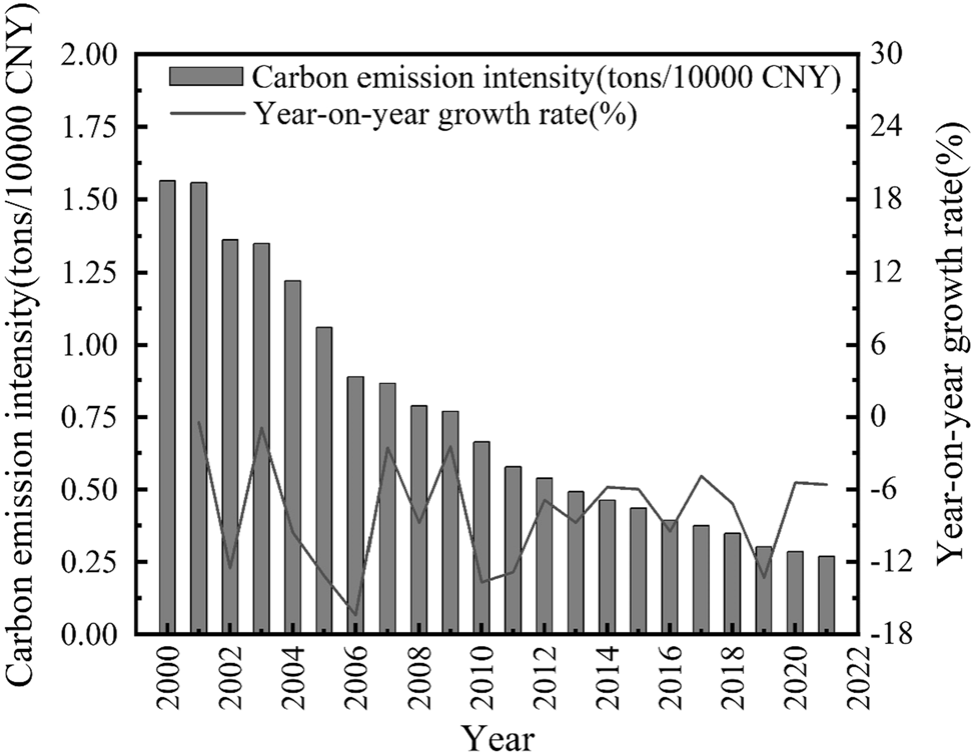
2020–2021 Agricultural carbon emission intensity and annual growth rate of Guangdong Province.

Agricultural carbon emissions and annual growth rates in Guangdong Province are shown in Fig. 1, and agricultural carbon emissions intensity and annual growth rates in Guangdong Province are shown in Fig. 2.

In a multiple linear regression model, multicollinearity is said to exist between explanatory variables if there is a close linear correlation between them. In cases of multicollinearity, the variance of the estimates of the regression parameters can be large, thus affecting the interpretation of the dependent variable by the independent variables, the precision of the estimates can be reduced and the estimates can become worse. Multicollinearity can be judged by constructing a new indicator tolerance $TOL$ (Tolerance) using the coefficient of determination, which is defined as:

7 $${TOL}_{(j)}= 1- {R}_{j}^{2} , j=\mathrm{1,2},\dots \dots ,m$$

where ${R}_{j}$ is the correlation coefficient between the independent variable ${X}_{j}$ and the other $m-1$ independent variables. If there is severe covariance between the independent variable ${X}_{j}$ and the other $m-1$ independent variables (i.e., ${R}_{j}^{2}$ ≈ 1), then ${TOL}_{(j)} \approx 0$. A tolerance value $TOL$ less than 0.2 can be considered as an indication of the presence of multicollinearity, and a tolerance value less than 0.1 indicates severe multicollinearity.

In order to solve the problem that the multiple linear regression model used in the STIRPAT model to construct an indicator system for the impact factors of agricultural emissions in Guangdong Province is prone to multiple co-linearity, this paper uses the Isomap algorithm to reduce the dimensionality of the data features. ISOMAP is a feature extraction-based dimensionality reduction algorithm, which is adapted from the multidimensional scaling algorithm (MDS), and its core idea is to use the "geodesic" distance instead of the "Euclidean distance" in MDS to calculate the distance between sample points. The core idea is to calculate the distance between sample points using 'geodesic' distances instead of 'Euclidean distances' in MDS. The original sample data of agricultural carbon emissions is fed into the Isomap algorithm for feature dimensionality reduction. The six new principal components ${t}_{1}$, ${t}_{2}$, ${t}_{3}$, ${t}_{4}$, ${t}_{5}$, ${t}_{6}$ are obtained by Isomap.

Next, the ACO-ET model will be used to predict agricultural carbon emissions in Guangdong Province using the new principal components after dimensionality reduction as input data.

This paper uses the ant colony algorithm (ACO) to find the optimal hyperparameters in the extreme random tree algorithm (ET). The two hyperparameters optimized in the extreme random tree algorithm (ET) are the maximum number of regression trees $n\_estimators$ and the maximum depth of each regression tree $max\_depth$, where the values of $n\_estimators$ are $\{100, 101, 102, 103, \dots \dots , 240\}$ , and $max\_depth$ are $\{1, 2, 3, 4,\dots \dots ,20\}$. A tenfold cross-validation was used to input the Guangdong agricultural carbon emission sample data into the model to find the optimal hyperparameters of the model and minimize the mean squared error index. The ACO-ET program was run and the optimal hyperparameters were: $n\_estimators$: 136, $max\_depth$: 6, which resulted in a minimum mean squared error of 0.0002.

The sample data of agricultural carbon emissions in Guangdong Province from 2000 to 2021 were taken as the data set and divided into a training set and a test set. Among them, the sample data from 2000 to 2018 are the training set and the data from 2019 to 2021 are the test set. The training set is used to train the combined prediction model Isomap–ACO–ET, and then the trained combined prediction model is used to test the test set, so as to evaluate the performance of the combined prediction model.

**Figure 3:**
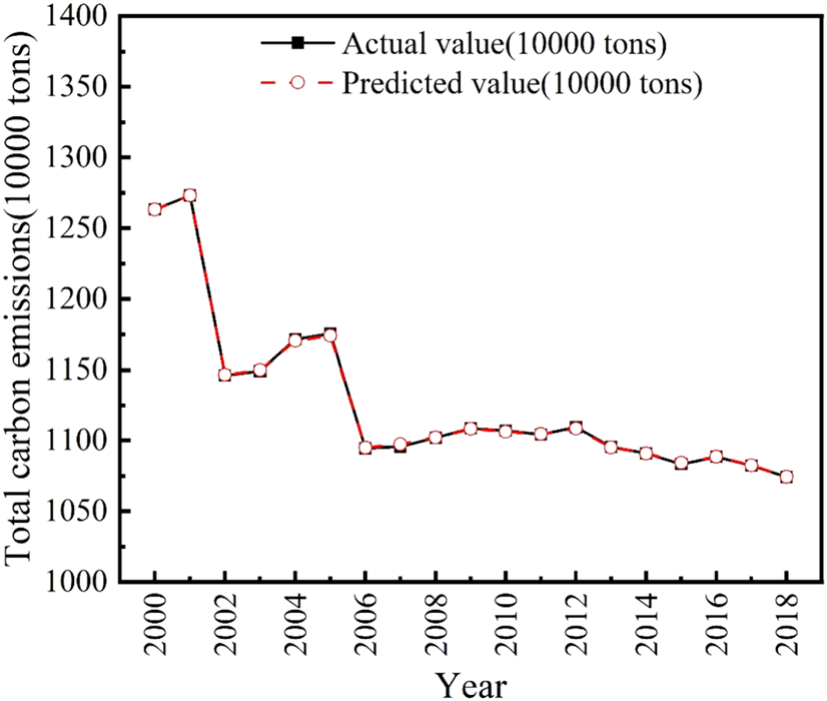
Training set fitting display.

A graph showing the fit of this combined prediction model to the training set is given in Fig. 3.

To verify the effectiveness of the Isomap–ACO–ET model proposed in this paper, which combines the isometric mapping algorithm (Isomap), the ant colony algorithm (ACO) and the extreme random tree algorithm (ET), more models were added for comparison. These include random forest algorithm (RF), gradient boosted tree algorithm (GBDT), recurrent neural network (RNN), and their models without Isomap algorithm but with ACO algorithm, with Isomap algorithm & ACO algorithm, with Principal component analysis & ACO algoritm, etc., and the metrics are evaluated for the performance using MSE, MAE, RMSE, MAPE. It can be found that the Isomap–ACO–ET model has the best performance evaluation metrics, indicating that the Isomap–ACO–ET combined forecasting model has the best forecasting effect. The comparative results of the performance evaluation of each model are shown in Table 1.

**Table 1:** Comparison result of all models in performance evaluation.

Model	MSE	MAE	RMSE	MAPE	Accuracy
ACO–RF	0.00022	0.0137	0.0148	0.1966	0.9798
Isomap–ACO–RF	0.00019	0.0130	0.0138	0.1860	0.9799
PCA–ACO–RF	0.00047	0.0199	0.0218	0.2850	0.9796
ACO–ET	0.00056	0.0231	0.0236	0.3310	0.9766
Isomap–ACO–ET	0.00018	0.0127	0.0136	0.1815	0.9873
PCA–ACO–ET	0.00043	0.0202	0.0208	0.2893	0.9796
ACO–GBDT	0.05987	0.2384	0.2447	33.0979	0.9214
Isomap–ACO–GBDT	0.00026	0.0157	0.0160	0.2247	0.9798
PCA–ACO–GBDT	0.00019	0.0129	0.0136	0.1845	0.9799
ACO–RNN	0.71738	0.6215	0.8470	97.6051	0.2770
Isomap–ACO–RNN	0.86195	0.6643	0.9284	209.0256	0.1353
PCA–ACO–RNN	1.30110	0.7784	1.1407	85.5976	0.0896

Based on the data of actual and predicted values for the period 2000–2021, we can calculate that the uncertainty of the combined prediction model is 8.238, which is 7.35 per cent of the average value and less than 10 per cent. It can be seen that the uncertainty is within the normal range, indicating that the model is reliable and credible.

Then, the trained Isomap–ACO–ET combined prediction model is applied to test the test set, and the results are shown in Table 2. It can be seen that the average prediction error rate of Isomap–ACO–ET combined prediction model is 0.013, and the predicted value is basically close to the actual value, which indicates that the prediction ability of Isomap–ACO–ET model is relatively good. Moreover, the Isomap–ACO–ET model has the smallest average error rate compared to both the ACO-ET without isometric mapping algorithm and the PCA-ACO-ET model using principal component analysis and ant colony algorithm. It can be seen that Isomap–ACO–ET has better prediction performance than the other two models, and the use of isometric mapping algorithm (Isomap) for dimensionality reduction of the data and the use of Ant Colony Algorithm (ACO) for optimization of the model parameters are beneficial to improve the prediction performance of the model.

**Table 2:** Comparative analysis of model prediction results.

Year	Actual value	Isomap–ACO–ET	Rate of error	ACO–ET	Rate of error	PCA–ACO–ET	Rate of error
2019	1064.205	1077.528	0.013	1092.159	0.026	1089.891	0.024
2020	1074.434	1081.773	0.007	1092.159	0.016	1088.396	0.013
2021	1063.121	1083.209	0.019	1092.159	0.027	1088.664	0.024

## Discussion

The next step is to explore the projected values of agricultural carbon emissions in Guangdong Province from 2022 to 2030. Different scenarios are set for the future of agriculture in Guangdong Province through the scenario analysis method. And the deductive analysis under different scenarios can make the possible future development scenarios clear and definite, which is helpful to provide a comprehensive reference for the prediction of agricultural carbon emissions in Guangdong Province under different scenarios.

Scenario analysis is a method to predict the possible situation or consequences of the predicted object under the premise that the trend of various indicator will continue into the future. This paper takes agriculture in Guangdong Province as the research object, and constructs the Isomap–ACO–ET combined prediction model of agricultural carbon emissions. In the model, the indicators of economic growth and technological progress that affect agricultural carbon emissions are considered, and the scenario analysis method is used to quantitatively predict the future carbon emission status of Guangdong agriculture under different development scenarios. In this paper, five different scenarios are set, which are maximum growth rate, average growth rate, minimum growth rate, positive minimum growth rate and positive average growth rate. For the parameters of different scenarios, please refer to the specific index data shown in Table 3 for details. The data are from the Guangdong Statistical Yearbook, cited from the website of the Guangdong Provincial Government of China.

**Table 3:** Indicator parameters under different scenarios.

Scenario	Rate	P	A	T	E	S	N	J
1	Max	0.0275	0.3633	0.0161	0.0748	0.0655	0.0608	0.1878
2	Pos-Min	0.0000	0.0087	0.0080	0.0045	0.0069	0.0113	0.0316
3	Average	−0.0222	0.1069	0.0056	0.0006	−0.0563	0.0301	0.0906
4	Pos-Ave	0.0101	0.1069	0.0116	0.0210	0.0265	0.0366	0.0906
5	Min	−0.1701	0.0087	−0.1136	−0.0732	−0.1542	−0.0995	0.0316

**Figure 4:**
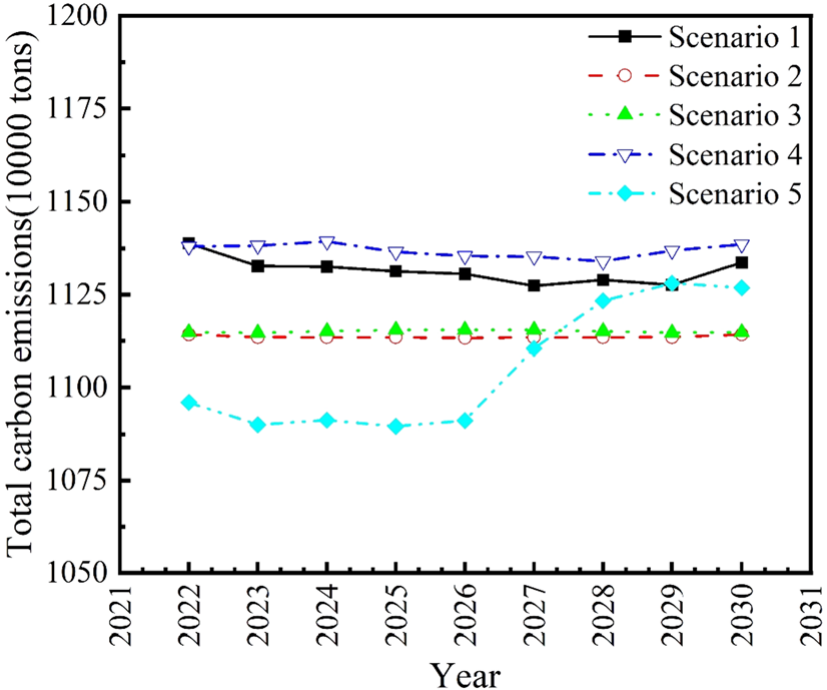
Prediction of agricultural carbon emissions in Guangdong Province from 2022 to 2030.

In this paper, different scenarios are set for the indicators affecting the prediction of agricultural carbon emissions in Guangdong Province, and the values of the indicators under each set scenario are predicted and the agricultural carbon emissions in Guangdong Province are predicted accordingly. Based on the above scenarios, the Isomap–ACO–ET model is used to predict the future agricultural carbon emissions in Guangdong Province under five scenarios: maximum growth rate, average growth rate, minimum growth rate, positive and minimum growth rate, and negative maximum growth rate. The predicting results of agricultural carbon emissions in Guangdong Province from 2022 to 2030 are shown in Fig. 4.

It can be seen from Fig. 4 that the predicted agricultural carbon emission of Guangdong Province in Scenario 1 shows a slow decline at first, from 11,388,200 tons in 2022 to 11,336,200 tons in 2030, a decrease of 0.45% in 9 years, with an average annual decline rate of 0.06%. In Scenario 2, the agricultural carbon emission of Guangdong Province is predicted to show a stable trend, from 11,142,200 tons in 2022 to 11,142,200 tons in 2030. In Scenario 3, the predicted agricultural carbon emission of Guangdong Province also shows a stable trend, from 11,148,000 tons in 2022 to 11,148,000 tons in 2030. In Scenario 4, the agricultural carbon emission of Guangdong Province is predicted to show a slight increase, from 11,380,100 tons in 2022 to 11,386,000 tons in 2030, with a slight increase of 0.05% in 9 years. In Scenario 5, the predicted agricultural carbon emissions in Guangdong Province shows an upward trend, rising from 10.960100 tons in 2022 to 11,268,700 tons in 2030, an increase of 2.82% in 9 years, with an average annual increase rate of 0.35%. In summary, the agricultural carbon emissions of Guangdong Province in China will fluctuate between 11,142,200 tons and 11,386,000 tons in 2030 according to the prediction under five different scenarios.

From the above analysis, it can be seen that in order to control agricultural carbon emissions in Guangdong Province in 2022–2030 at a lower growth level, Guangdong Province should try to develop its agricultural industry according to scenarios such as Scenario 2 and Scenario 3, i.e. That is, the seven indicators such as agricultural production efficiency and agricultural industry structure should be controlled under the development level of positive and minimum growth rate and average growth rate, so as to effectively control the future carbon emission discharge of Guangdong Province’s agriculture and make Guangdong Province’s agriculture to be able to develop in a green environmentally friendly and healthy way.

Agriculture in Guangdong Province is in a period of accelerated urbanization and industrialization, with large quantities of “three wastes (waste gas, waste water, waste residue)” emitted from urban life and industrial production. The heavy use of chemical fertilizers and pesticides in the agricultural production process has resulted in serious surface and endogenous pollution, which has damaged the rural ecosystem and the biological chain, leading to a decrease in the number of biological species and the contamination of the air and water systems^33^. Guangdong has an advantage in terms of economic volume and scale, with its economic volume accounting for about one-eighth of the country’s total over the years, and its strong economic scale and economic strength has laid the economic foundation for the development of low-carbon rural areas and the restructuring of the agricultural economy^34^. Guangdong Province should improve farming practices, reduce the intensity of agrochemical application, increase the level of soil carbon sequestration, and gradually increase the proportion of organic fertilizers, bio-pesticides and other low-carbon green production materials used to promote soil nutrient balance and curb the loss of soil organic carbon and greenhouse gas emissions^35^. At the same time, we should strengthen the development and promotion of agricultural energy-saving and emission reduction technologies and low-carbon energy sources, improve the efficiency of energy use, vigorously develop and use rural renewable energy sources, optimize the energy consumption structure of agricultural production, and reduce the carbon emission intensity of agricultural energy consumption^36^. In addition, the agricultural planting structure should be optimized, and crops such as southern subtropical fruits, vegetables and flowers with Guangdong characteristics and competitive advantages should be developed according to local conditions, so as to build an industrial belt of advantageous agricultural products and improve the quality and efficiency of agricultural products^37^. Once again, leisure tourism and eco-tourism agriculture should be developed to promote the improvement of the rural ecological environment in order to ensure the healthy and sustainable development of the agricultural ecology of Guangdong Province^38^.

## Conclusion

The scientific analysis of regional agricultural carbon emission prediction models and empirical studies are of great practical significance for China to achieve low-carbon agriculture, implement the strategy of rural revitalization, and build an ecologically civilized and beautiful countryside. In order to better empirically study the regional agricultural carbon emission problem, this paper takes the agriculture of Guangdong Province, China, as the research object, constructs a combined Isomap–ACO–ET prediction model based on Isomap, ACO and ET. And the indicators of factors affecting agricultural carbon emissions, such as agricultural population, agricultural industrial structure, production efficiency, etc. were selected to predict the agricultural carbon emissions in Guangdong Province under five scenarios. The results show that this method can effectively predict agricultural carbon emissions in Guangdong Province, which are expected to fluctuate between 11,142,200 tons and 11,386,600 tons in 2030. And compared with other machine learning and neural network models, the Isomap–ACO–ET model has better prediction performance with MSE of 0.00018 and accuracy rate of 98.7%. The policy recommendations for the development of low-carbon agriculture in Guangdong Province are as follows: Firstly, improve farming practices, reduce the intensity of agrochemical application and increase the level of soil carbon sequestration. The main focus is on transforming traditional farming methods and implementing conservation tillage, as well as improving the efficiency of the use of high-carbon-based production materials such as chemical fertilizers and pesticides, and reducing the intensity of their application. Secondly, strengthen the development and promotion of agricultural energy-saving and emission reduction technologies and low-carbon energy sources, and reduce the intensity of carbon emissions from agricultural energy consumption. The main focus is to strengthen the development and promotion of agricultural energy conservation and emission reduction technologies and improve the efficiency of energy use. As well as vigorously developing and utilizing rural renewable energy sources and optimizing the structure of energy consumption in agricultural production. Thirdly, optimize the structure of agricultural cultivation and develop green agricultural products and agroecological tourism in accordance with local conditions.

This paper has conducted an empirical study on agricultural carbon emissions in Guangdong Province, but there are still some areas that need further research and improvement. At present, there are many influencing factors involved in the research due to agricultural carbon emissions. Apart from economic growth and technological progress, other aspects such as local policies and national policies will also have an impact. In the future, the scope of research can be expanded to address the impact of regulations and policies on agriculture, and the regional characteristics of Guangdong Province can be analyzed to further study the situation of agricultural carbon emissions in Guangdong Province.

## Data Availability

The datasets used and/or analyzed during the current study are available from the corresponding author on reasonable request.
